# Desmoplastic fibroma with perineural spread: conventional and diffusion-weighted magnetic resonance imaging findings

**DOI:** 10.1590/0100-3984.2014.0135

**Published:** 2015

**Authors:** Bruno Niemeyer de Freitas Ribeiro, Tiago Medina Salata, Lívia de Oliveira Antunes, Edson Marchiori

**Affiliations:** 1 Instituto Estadual do Cérebro Paulo Niemeyer, Rio de Janeiro, RJ, Brazil.; 2 Hospital Casa de Portugal / 3D Diagnóstico por Imagem, Rio de Janeiro, RJ, Brazil.; 3 Universidade Federal do Rio de Janeiro (UFRJ), Rio de Janeiro, RJ, Brazil.

*Dear Editor*, 

A male, three-year-old child with morphostructural alteration developed over the last year in the region of the mandible at left, presenting with recent onset of pain, with no other associated complaints. Laboratory tests did not demonstrate any alteration and magnetic resonance imaging (MRI) (Figure 1) showed a lesion with predominant iso/hyposignal on T1-weighted image, hypersignal on T2-weighted image with subtle low signal intensity foci, absence of signal loss on susceptibility-weighted sequences and absence of diffusion restriction. After gadolinium injection, exuberant enhancement was observed in addition to perineural dissemination through the third division of the trigeminal nerve. Histopathological analysis revealed spindle cells without atypias and pleomorphism, besides areas with acellular fibrous connective tissue, with immunohistochemical negative for S100, and positivity for vimentin and SMA, with Ki-67 < 5%. Such findings are compatible with desmoplastic fibromas. The patient was submitted to incomplete surgical excision supplemented with radiotherapy.

**Figure 1:**
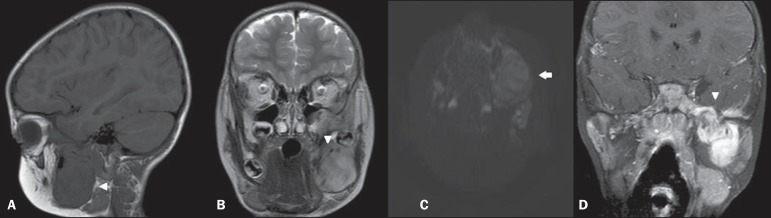
**A:** Sagittal, T1-weighted image showing lesion with hyposignal affecting the mandible (arrowhead). **B:** Coronal, T2-weighted sequence showing heterogeneous lesion with subtle hypersignal intermingled with foci of low signal intensity (arrowhead). **C:** Axial, functional diffusion-weighted sequence does not demonstrate diffusion restriction (arrow). **D:** Contrast-enhanced coronal, T1-weighted sequence with fat suppression demonstrating exuberant gadolinium enhancement and noticeable perineural dissemination in the third division of the trigeminal nerve (arrowhead).

Desmoplastic fibroma is an extremely rare, benign bone tumor with aggressive and usually insidious behavior, representing 0.1% of all primary bone tumors^(1-5)^. The mandible is the most affected site, particularly in its posterior portion, corresponding to 22% of cases^(1,2,4)^, followed by the metaphyseal region of long bones. Desmoplastic fibromas may occur at any age range, although its higher incidence is observed at the first three decades of life^(1-3,6)^. Despite conflicting data, it seems there is no predilection for sex^(2,6)^. Local recurrence is frequently observed in cases where complete resection is not. Clinically, the patients are either asymptomatic or may present with pain, edema, joint effusion and pathological fracture^(1-6)^. The differential diagnosis should consider rhabdomyosarcoma, fibrosarcoma, giant cell tumor, among others. Despite the imaging methods usefulness in the lesion delimitation, the diagnosis is histopathological.

At MRI, most lesions present with iso/hyposignal on T1weighted images and low signal intensity on T2-weighted images^(1,3-6)^, but there are reports of lesions with hypersignal on T2weighted images^(1-3,6)^. The enhancement may be variable, and according to some authors, such variation may be a result of the cellular content of the lesion^(3,4)^. In the present case, there was homogeneous iso/hyposignal on T1-weighted images and subtle hypersignal on T2-weighted images, with foci of low signal intensity. After gadolinium injection, marked contrast enhancement, with noticeable perineural dissemination through the third division of the trigeminal nerve were observed. Such aspects on T2weighted sequences, and the presence of perineural dissemination are not commonly observed as compared with the typical imaging pattern described at MRI.

Reports on diffusion in desmoplastic fibromas were not found in the literature. In the present case, areas of diffusion restriction were not observed. Recent studies highlight the use of diffusionweighted imaging in the evaluation of head and neck lesions, showing that apparent diffusion coefficient < 1.22 × 10^–3^ mm^2^/s are suggestive of malignancy^(7)^. In the present case, the value for apparent diffusion coefficient was 1.45 × 10^–3^ mm^2^/s, corroborating the previously described findings.

The authors conclude that the diagnosis of desmoplastic fibromas should be considered in patients under the age of 30 presenting with tumor particularly located in the mandible, and that such a hypothesis cannot be ruled out in case of less noticeable foci of hyposignal on T2-weighted images.

## References

[1] Woods TR, Cohen DM, Islam MN (2015). Desmoplastic fibroma of the mandible: a series of three cases and
review of literature. Head Neck Pathol.

[2] Nedopil A, Raab P, Rudert M (2013). Desmoplastic fibroma: a case report with three years of clinical and
radiographic observation and review of the literature. The Open Orthopaedics Journal.

[3] Kim OH, Kim SJ, Kim JY (2013). Desmoplastic fibroma of bone in a toe: radiographic and MRI
findings. Korean J Radiol.

[4] Kang DM, Juhng SK, Sohn YJ (2014). Imaging findings of desmoplastic fibroma rarely involving the
clavicle: case report. Korean J Radiol.

[5] Frick MA, Sundaram M, Unni KK (2005). Imaging findings in desmoplastic fibroma of bone: distinctive T2
characteristics. AJR Am J Roentgenol.

[6] Moorjani V, Stockton V (2005). Desmoplastic fibroma with perineural extension. AJR Am J Roentgenol.

[7] Gonçalves FG, Ovalle JP, Grieb DFJ (2011). Diffusion in the head and neck: an assessment beyond the
anatomy. Radiol Bras.

